# A scoping review of physical activity interventions for older adults

**DOI:** 10.1186/s12966-021-01140-9

**Published:** 2021-06-30

**Authors:** Jennifer Taylor, Sarah Walsh, Wing Kwok, Marina B. Pinheiro, Juliana Souza de Oliveira, Leanne Hassett, Adrian Bauman, Fiona Bull, Anne Tiedemann, Catherine Sherrington

**Affiliations:** 1 Institute for Musculoskeletal Health, The University of Sydney and Sydney Local Health District, Camperdown, NSW 2050 Australia; 2 School of Public Health, Faculty of Medicine and Health, The University of Sydney, Sydney, Australia; 3 Discipline of Physiotherapy, Sydney School of Health Sciences, Faculty of Medicine and Health, The University of Sydney, Sydney, Australia; 4 Charles Perkins Centre, School of Public Health, Faculty of Medicine and Health, The University of Sydney, Sydney, Australia; 5 WHO Collaborating Centre for Physical Activity, Nutrition and Obesity, Sydney, Australia; 6 Physical Activity Unit, Department of Health Promotion, Division of Universal Health Coverage and Healthier Populations, World Health Organization (WHO), Geneva, Switzerland

**Keywords:** Aged, Exercise, Healthy aging, Older adults, Physical activity, World Health Organization

## Abstract

**Background:**

To inform implementation and future research, this scoping review investigates the volume of evidence for physical activity interventions among adults aged 60+. Our research questions are: (1) what is the evidence regarding interventions designed to increase total physical activity in adults aged 60+ years, in accordance with three of the four strategic objectives of GAPPA (active societies, active environments, active people); (2) what is the current evidence regarding the effectiveness of physical activity programmes and services designed for older adults?; and (3) What are the evidence gaps requiring further research?

**Methods:**

We searched PEDro, MEDLINE, CINAHL and Cochrane from 1 January 2010 to 1 November 2020 for systematic reviews and meta-analyses of physical activity interventions in adults aged 60+. We identified interventions designed to: (1) increase physical activity; and (2) deliver physical activity programmes and services in home, community or outpatient settings. We extracted and coded data from eligible reviews according to our proposed framework informed by TIDieR, Prevention of Falls Network Europe (PROFANE), and WHO’s International Classification of Functioning, Disability and Health (ICF). We classified the overall findings as positive, negative or inconclusive.

**Results:**

We identified 39 reviews of interventions to increase physical activity and 342 reviews of programmes/services for older adults. Interventions were predominantly structured exercise programmes, including balance strength/resistance training, and physical recreation, such as yoga and tai chi. There were few reviews of health promotion/coaching and health professional education/referral, and none of sport, workplace, sociocultural or environmental interventions. Fewer reported outcomes of total physical activity, social participation and quality of life/well-being. We noted insufficient coverage in diverse and disadvantaged samples and low-middle income countries.

**Conclusions:**

There is a modest but growing volume of evidence regarding interventions designed to increase total physical activity in older adults, although more interventional studies with long term follow-up are needed, particularly for GAPPA 1. Active Societies and GAPPA 2. Active Environments. By comparison, there is abundant evidence for GAPPA 3. specific programmes and services, but coverage of sport and workplace interventions, and diverse samples and settings is lacking. Comprehensive reviews of individual studies are now needed as well as research targeting neglected outcomes, populations and settings.

## Background

In October 2020 the World Health Organization (WHO) launched the Decade of Healthy Ageing 2021–2030 in response to rapid, global population ageing [1]. There are currently more than one billion people (12%) aged over 60 years and this is forecast to grow to 2 billion (22%) by 2050 [2]. Moreover, 80% of these older adults are projected to live in low- and middle-income countries with limited access to health services and, as shown by the current COVID-19 pandemic, age is associated with increased risk of disease, co-morbidity and loss of independence [2]. This initiative is part of the United Nations’ 2030 Sustainable Development Agenda [3] and WHO Global Strategy and Action Plan on Ageing and Health [2] (GSAP) and argues a universal right to health at all ages.

Once called the ‘Cinderella risk factor’ for non-communicable disease (NCD) prevention due to policy neglect and inadequate resourcing [4], physical activity is globally recognised as important for supporting healthy ageing in a number of ways. In the 2015 *Report on Ageing and Health* [5] WHO identified the potential for physical activity to slow age-related decline in functional ability, and develop and maintain physical and mental intrinsic capacity in older adults. This new conceptual approach acknowledges the functional diversity among older adults and focuses on health and capability rather than chronological age. In this way, physical activity is a key enabler of work, social contribution, autonomy and dignity as well as health in older age.

The 2020 *WHO Guidelines on Physical Activity and Sedentary Behaviour* [6] call for older adults to undertake 150–300 min of moderate intensity physical activity (or 75–150 min vigorous), including muscle strengthening on at least 2 days per week and varied multicomponent physical activity emphasizing functional balance and strength training on at least 3 days per week. There is an urgent need for global action on physical inactivity as one in four adults are insufficiently active with higher rates in women and older adults [7]. Only one in two older adults regularly undertake physical activity and fewer engage in multicomponent physical activity targeting functional balance and strength as recommended in the 2020 *WHO Guidelines* [6]. The WHO *Global Action Plan on Physical Activity* (GAPPA) sets out four strategic policy objectives (Active Societies, Active Environments, Active People, and Active Systems) and 20 related policy actions, that aim to address the social, cultural, environmental and individual determinants of physical inactivity. The resolution adopting the GAPPA (World Health Assembly WHA71) also recorded member states’ request for support with implementation, and specific tools and resources for the policy actions areas.

To assist countries to adopt, tailor and implement the recommendations into their local contexts, WHO is developing ACTIVE [8], a technical toolkit with implementation guidance for key approaches, settings and population groups. WHO commissioned this scoping review of physical activity intervention literature for older adults to assess available volume of evidence to support toolkit development and inform the direction of further research.

Previous reviews of physical activity literature for older adults focus largely on falls prevention [9], acute care rehabilitation, and/or specific health conditions [10, 11]. These condition-centric approaches are useful for clinical practice but sub-optimal to inform population-wide intervention approaches. There is one recent notable exception in Di Lorito et al. [12], although this review targeted structured exercise rather than the wider definition of physical activity and/or alternate approaches to increase physical activity. There are also some scoping reviews of physical activity in older adults [13, 14], although none attempt a public health approach. Given our interest in interventions to: [1] increase total physical activity; and [2] deliver physical activity programmes and services for older adults, we have chosen a scoping review methodology to deliver a timely, initial assessment of the entire physical activity evidence base.

### Objectives

We conducted a scoping review of systematic reviews and meta-analyses of physical activity interventions for older adults and classified them using a two-component conceptual framework that we developed based on the GAPPA, the *International Classification of Functioning, Disability and Health* (ICF) [15]*,* the Consolidated Standards of Reporting Trials (CONSORT) Template for Intervention Description and Replication (TIDieR) framework [16, 17] and the Prevention of Falls Network Europe (PROFANE) taxonomy [18, 19].

This scoping review of physical activity interventions for older adults addresses the following research questions:

1. What is the evidence regarding interventions designed to *increase total physical activity* in adults aged 60+ years, and *how might they be classified* within three of the four strategic objectives of GAPPA:
  - Active societies
  - Active environments
  - Active people
2. What is the current evidence regarding effectiveness for GAPPA Action 3.4 to *deliver physical activity programmes and services,* and:

*“Enhance the provision of, and opportunities for, appropriately tailored programmes and services aimed at increasing physical activity and reducing sedentary behaviour in older adults, according to ability, in key settings such as local and community venues, health, social and long-term care settings, assisted living facilities and family environments, to support healthy ageing.”?* [7]

3. What are the evidence gaps requiring further research?

Research question one includes a broad range of interventions that aim to increase total/overall physical activity in older adults. These may include: physical activity promotion in mass media campaigns; health professional coaching/referral schemes; environmental programs and/or community campaigns; as well as structured exercise or physical activity programmes and devices with total physical activity outcomes. Research question two focuses on interventions that deliver exercise and physical activity programmes directly to older adults and consider the impact on a range of outcomes. These often incorporate outcomes of strength, balance, or mobility, and may/may not measure total physical activity.

## Method

This scoping review was conducted in accordance with the *PRISMA Extension for Scoping Reviews* (PRISMA-ScR) [20]. A protocol was prepared in advance and published on the *Open Science Framework* [21].

### Search strategy

We searched the following four databases: *Physiotherapy Evidence Database (PEDro), MEDLINE*, *Cumulative Index to Nursing and Allied Health Literature (CINAHL)*, and the *Cochrane Database of Systematic Reviews* from 1 January 2010 to 1 November 2020. Keywords, MeSH and other index terms, as well as combinations of these terms and appropriate synonyms, were used to construct the search strategy. Titles and abstracts were screened independently by two reviewers (JT and SW) using Covidence systematic review software, Veritas Health Innovation, Melbourne, Australia. Four reviewers read full-texts and assessed eligibility criteria (JT, SW, JSO, MP). Discrepancies were resolved by consensus or referred to senior investigators (CS and AT). A sample search strategy is included at Table 1.

**Table 1:** Sample MEDLINE search strategy

Description	Search terms
Limit: language and exclude animal only	(English[lang]) NOT (“Animals”[Mesh] NOT (“Animals”[Mesh] AND “Humans”[Mesh]))
Population	AND “old”[tiab] OR “older”[tiab] OR “aged”[tiab] OR “aging”[tiab] OR “ageing”[tiab] OR “elderly” [tiab]
Limit: age groups	NOT ((“infant”[Mesh] OR “child”[mesh] OR “adolescent”[mh]) NOT ((“infant”[Mesh] OR “child”[mesh] OR “adolescent”[mh]) AND “adult”[Mesh]))
Limit: date	AND (“2010/01/01”[PDAT]: “3000/12/31”[PDAT])
Publication type	AND (systematic[sb] OR meta-analysis[pt] OR “systematic review” [tiab] OR “systematic literature review” [tiab] OR metaanalysis[tiab] OR “meta analysis”[tiab] OR metanalyses[tiab] OR “meta analyses”[tiab] OR “pooled analysis”[tiab] OR “pooled analyses” [tiab] OR “pooled data”[tiab])
Limit: publication type	NOT (“comment” [Publication Type] OR “editorial” [Publication Type])
Physical activity:	AND ((“Activities of daily living”[tiab] OR “Activity of daily living”[tiab] OR “Aerobic activities”[tiab] OR “Aerobic activity”[tiab] OR “Balance training”[tiab] OR “Cardiovascular activities”[tiab] OR “Cardiovascular activity”[tiab] OR “Chi kung”[tiab] OR “Endurance activities”[tiab] OR “Endurance activity”[tiab] OR “Exercise”[mh] OR “Exercise”[tiab] OR “Free living activities”[tiab] OR “Free living activity”[tiab] OR “Functional training”[tiab] OR “Lifestyle activities”[tiab] OR “Lifestyle activity”[tiab] OR “Physical activity”[tiab] OR “Qigong”[tiab] OR “Recreational activities”[tiab] OR “Recreational activity”[tiab] OR “stretching”[tiab] OR “Tai ji”[mh] OR “Yoga”[mh] OR “Qigong”[mh]) OR “Physical activities”[tiab] OR “Physical conditioning”[tiab] OR “Resistance training”[tiab] OR “strength training”[tiab] OR “Tai chi”[tiab] OR “Tai ji”[tiab] OR “Walk”[tiab] OR “Walking”[tiab] OR “Yoga”[tiab] NOT medline[sb]))

### Eligibility criteria

Studies were selected for research questions one and/or research question two according to eligibility criteria described in Table 2. In general, studies were eligible if they reported on interventions that aimed to increase overall physical activity and addressed any of the first three GAPPA objectives: Active Societies; Active Environments; and Active People. GAPPA objective four, Active Systems, was excluded during study design given this applies predominantly to organising policy and legislative frameworks not customarily returned by the search terms and databases in scope.

**Table 2:** Review eligibility criteria

	Research Question 1		Research Question 2	
	Interventions to increase physical activity in older adults		Physical activity programmes & services for older adults	
	Inclusion	Exclusion	Inclusion	Exclusion
Population	Adults aged 60 years and older both sexes not selected for pre-existing medical conditions. Mixed heathy and chronic conditions (e.g. sarcopenia, osteopenia, obesity, mild cognitive impairment)	Total average age less than 60 years Total sample selected for specific medical conditions (e.g. stroke, dementia, Parkinson’s disease)	Adults aged 60 years and older both sexes not selected for pre-existing medical conditions. Mixed healthy and chronic conditions (e.g. sarcopenia, osteopenia, obesity, mild cognitive impairment)	Total average age less than 60 years Total sample selected for specific medical conditions
Intervention	Any intervention aimed at increasing total physical activity, including environment interventions and health professional/ referral schemes.	Studies of single sessions of physical activity were excluded	Any physical activity programme or service aimed at older adults	Studies of single sessions of physical activity were excluded Multicomponent interventions such as nutrition and exercise. General occupational therapy/physiotherapy
Comparison	Any comparator		Any comparator	
Outcomes	Any longitudinal total physical activity outcome (e.g. MVPA^a^, steps, energy expenditure, physical activity questionnaire)	Adherence only Barriers/facilitators only	Any outcome of interest (physical activity, social, physical, cognitive/emotion functioning, and overall well-being /quality of life)	Adherence only Barriers/facilitators only
Settings	Recruitment in all settings Intervention delivery in clinical outpatient, residential care, community/home settings	Hospital/clinical inpatient settings	Recruitment in all settings Intervention delivery in clinical outpatient, residential care, community/home settings	Hospital inpatient settings
Study design	Systematic reviews and meta-analyses of interventional studies	Meta-reviews, reviews of reviews	Systematic reviews and meta-analyses of interventional studies	Meta-reviews, reviews of reviews

^a^*MVPA* moderate-vigorous physical activity usually measured in minutes/week

We only included systematic reviews and meta-analyses of interventional studies. Individual studies and comparators did not form part of review selection criteria, although systematic reviews of individual studies may have included controlled and uncontrolled trials. Systematic reviews of cross-sectional and other observational studies were excluded, as were reviews of short-term/acute, and major, multicomponent interventions. Reviews were also excluded if they targeted specific medical conditions or diagnoses. Review quality was not assessed in this scoping review.

### Data extraction, synthesis, and charting

Four independent reviewers (JT, SW, WK, LH) extracted the data using a standardised electronic data extraction form. The following data were extracted from each review: author; publication year; country; number of eligible trials; total participants; average age; and overall findings effect indicator (positive, negative or inconclusive) [22]. Our approach to effect indicator coding was generous in that we recorded a positive result where authors reported at least one outcome in our domains of interest in favour of the review intervention(s). A positive result for the purpose of this scoping review does not necessarily mean statistical significance in meta-analysis or at the level of each individual study.

Reviews were then coded according to the GAPPA objectives for research question one and a proposed two-tier classification framework for research question two. These were based on: population; intervention (informed by the TIDieR framework and PROFANE taxonomy); comparator; and outcome (PICO, informed by the ICF). Reviews eligible for research question two were coded according to framework classification level 2 (Table 3). We first coded the reviews with all (100%) of studies in a particular category (Table 3, column 4). However, given the large proportion of reviews with ‘mixed’ category results, we also coded reviews that included any (1+) studies in that category (Table 3, column 5).

**Table 3:** Systematic reviews of physical activity programmes for older adults

PICO/ TIDieR item	PICO aspect of interest, adaption of TIDieR	Framework classification level 1	Number of reviews with ALL studies in category^a^ k/342 (%)	Number of reviews with ANY (1+) studies in category^b^ k/342 (%)
Population (Older adults)	Country	High-income	126 (37%)	327 (96%)
		Upper middle income	3 (1%)	201 (59%)
		Lower-middle income	0	15 (4%)
		Low income	0	0
		Mixed	213 (62%)	–
	Particular groups	Care facility residents	15 (4%)	95 (28%)
		Own home/community	245 (72%)	327 (96%)
		“Older” old age	1 (< 1%)	–
		Impaired capacity	17 (5%)	166 (49%)
		Health conditions	6 (2%)	194 (57%)
		Rural/regional	1 (< 1%)	–
		Cultural or indigenous background	1 (< 1%)	–
		Socio-economic status	3 (1%)	26 (8%)
	Gender	Male	0 (0%)	341 (99%)
		Female	1 (< 1%)	342 (100%)
		Any	341 (99%)	–
Intervention	Type of programme	Physical activity delivered/mixed	331 (97%)	342 (100%)
		Physical activity promoted	11 (3%)	49 (14%)
	Type of physical activity	Total activity	11 (3%)	49 (14%)
		Structured exercise	243 (71%)	298 (87%)
		Recreation	20 (6%)	70 (20%)
		Sport	0 (0%)	3 (1%)
	Provider	Professional	297 (87%)	339 (99%)
		Volunteer	0 (0%)	8 (2%)
		Carer	0 (0%)	4 (1%)
		None	0 (0%)	35 (10%)
		Mixed	45 (13%)	–
	Who with	Individual	81 (24%)	291 (85%)
		Group	46 (13%)	255 (75%)
		Mixed	215 (63%)	–
	Delivery mode	Synchronous/ live	239 (70%)	338 (99%)
		Asynchronous/ pre-recorded	3 (1%)	103 (30%)
		Mixed	100 (29%)	–
	Location	Health service	8 (2%)	100 (29%)
		Workplace	0 (0%)	3 (1%)
		Community facility	57 (17%)	283 (83%)
		Residential care	9 (3%)	72 (21%)
		Assisted living	0 (0%)	11 (3%)
		Own home	8 (2%)	239 (70%)
		Faith-based	0 (0%)	1 (< 1%)
		Park/ sports field	1 (< 1%)	109 (32%)
		Mixed	259 (76%)	–
Comparison	No intervention	No intervention	108 (32%)	332 (97%)
	Physical activity	Higher dose of same activity	0 (0%)	0 (0%)
		Different physical activity	8 (2%)	171 (50%)
	Other intervention	Education	0 (0%)	130 (38%)
		Other	0 (0%)	86 (25%)
	Mixed	Mixed	225 (66%)	–
Outcome	Physical activity	Self-report	4 (1%)	36 (11%)
		Observation	10 (3%)	41 (12%)
		Device-based	4 (1%)	29 (8%)
		Mixed	37 (11%)	–
		None	287 (84%)	–
	Social functioning (participation)	Self-report	6 (2%)	13 (4%)
		Observation	4 (1%)	10 (3%)
		Device-based	0 (0%)	4 (1%)
		Mixed	5 (1%)	–
		None	326 (95%)	–
	Physical functioning	Self-report	2 (1%)	45 (13%)
		Observation	74 (22%)	177 (52%)
		Device-based	25 (7%)	121 (35%)
		Mixed	148 (43%)	–
		None	93 (27%)	–
	Cognitive and emotional functioning	Self-report	21 (6%)	27 (8%)
		Observation	72 (21%)	95 (28%)
		Device-based	2 (1%)	14 (4%)
		Mixed	28 (8%)	–
		None	219 (64%)	–
	Well-being, quality of life, composite measures of functioning	Self-report	26 (8%)	–
		Observation	48 (14%)	–
		Mixed	10 (3%)	–
		None	258 (75%)	–

^a^This column indicates reviews with ALL included studies in this category. That is, the subcategories are mutually exclusive and the total of categories equals the 342 included reviews

^b^This column shows reviews that included ANY studies meeting this category definitions. That is, the subcategories are not mutually exclusive and one review may be reported in multiple subcategories

Data coding, aggregation and charting was performed in Microsoft Excel using extracted data and framework coding.

## Results

**Fig. 1:**
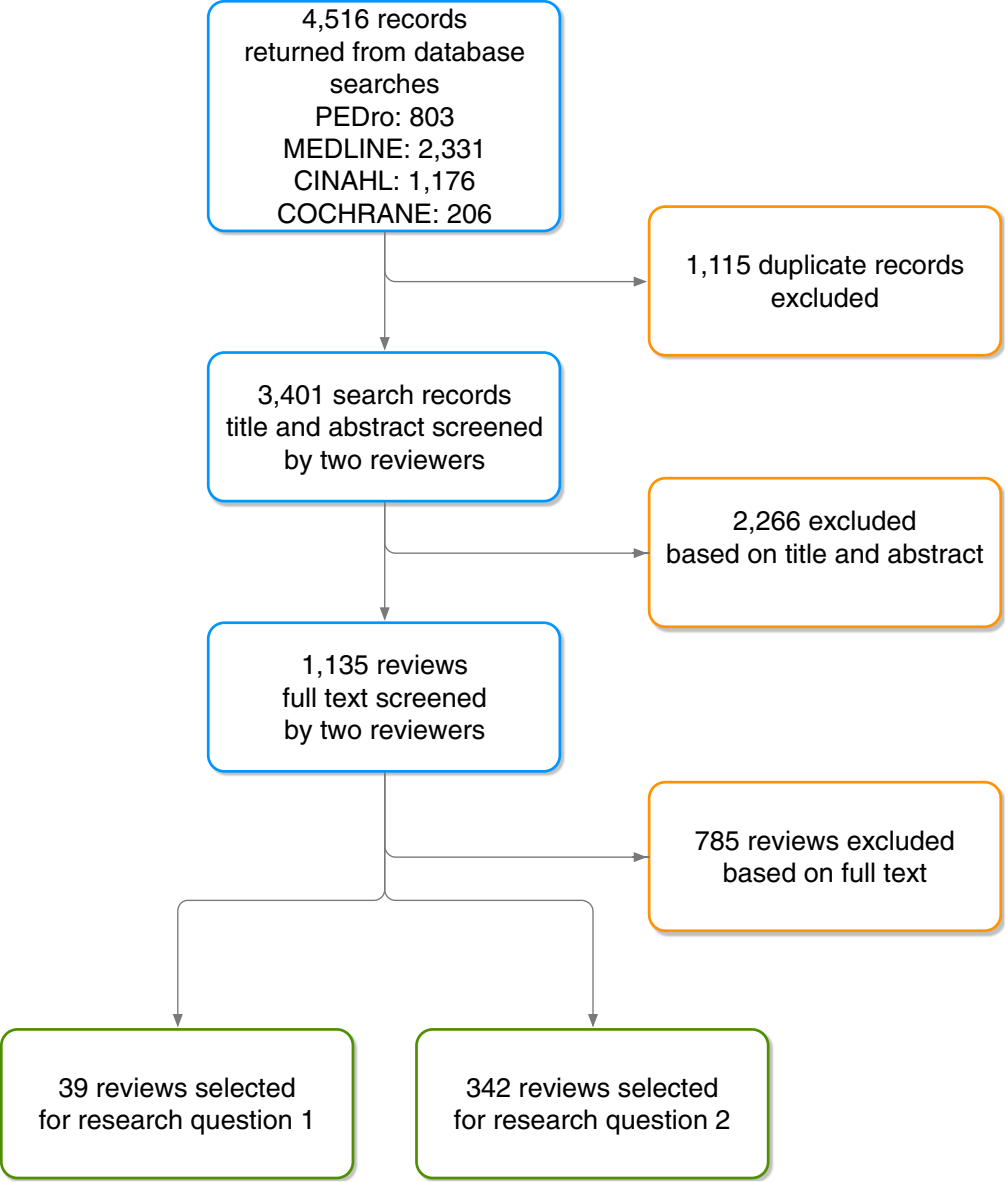
PRISMA-ScR flow diagram

Our searches returned 4516 records. After deduplication, two reviewers screened 3401 articles by title and abstract, then full text of 1135 articles, leaving a final 39 reviews eligible for research question one and 342 reviews for research question two. We documented the screening process in a PRISMA-ScR study flow diagram (Fig. 1).

**Fig. 2:**
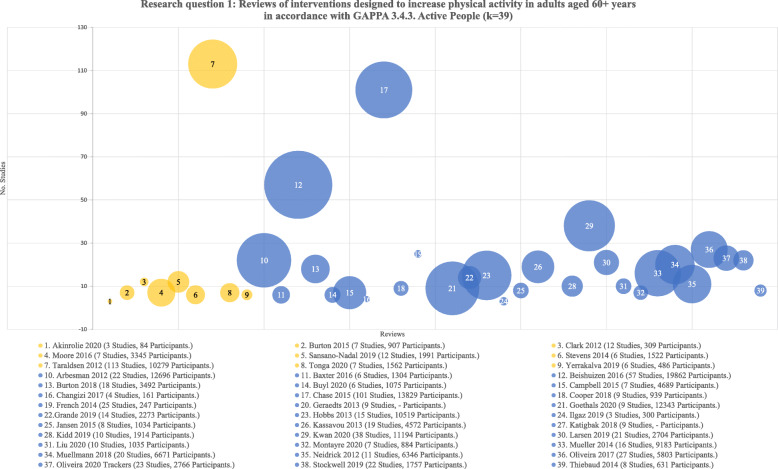
Research question one – chart of reviews by number of studies reviewed and total participants. Note: Given all reviews for research question one were plotted (k = 39), the X-axis was removed as it represents sequential numbering only. The Y axis represents the number of studies in each review and bubble size indicates the total number of participants (where available). Total n was not calculated as studies may be in multiple reviews [23–60]

Reviews eligible for research question one focused on interventions to increase total/overall physical activity in older adults. We identified 39 such reviews, 30 with overall positive and nine with inconclusive findings. Between 2012 and 2017, three reviews were published per year on average, increasing to eight reviews per year on average between 2019 and 2020. The most common types of interventions were health promotion, eHealth, and activity trackers. As seen in Fig. 2, the number of studies in each review ranged between 3 and 113 studies (M = 10) based on database searches between 1980 and 2019. Of those with positive findings (i.e. increased total physical activity post-intervention), the number of studies ranged between 3 and 101 studies (M = 13). The number of participants ranged between 84 [23] and 19,862 [24] with a median age of 67 years. The number of studies and/or participants were not further aggregated in this analysis because studies may appear in more than one review.

All reviews were classified as *GAPPA Objective 3. Active People* (39 reviews) with no reviews addressing *1. Active Societies* or *2. Active Environments*. The majority (30/39, 77%) of the reviews in *3. Active People* reported positive findings, in favour of intervention(s), for programmes resulting in increased overall physical activity in older adults while the remaining nine (9/39, 23%) were inconclusive.

Included reviews were generally authored in high-income countries: the UK (9/39, 23%), Australia (7/39, 18%), the United States (5/39, 13%), China (2/39, 5%), other European countries (9/39, 23%) and Canada (1/39, 2.5%). There were also reviews (one each) from Brazil, Colombia, Iran, Malaysia, and Turkey (2/39, 5%).

Reviews eligible for research question two focused on specific programmes and services of physical activity delivered directly to older adults. We identified 342 reviews that included outcomes of interest other than total/overall physical activity, such as physical functioning. Between 2010 and 2012, 14 of these reviews were published per year on average, increasing to 55 per year on average 2019–2020.

Reviews included studies from high- and upper-middle income countries almost exclusively. We found no reviews with studies from discernibly low-income countries. Samples were mostly community-dwelling older adults with some studies in residential care (Table 3), particularly of falls. There was little coverage of vulnerable populations, such as the ‘oldest’ old, rural/regional or migrant/indigenous backgrounds. While we actively excluded reviews with health conditions as eligibility criteria, more than half the reviews included studies that reported on mixed samples with some health conditions/risk factors.

**Fig. 3:**
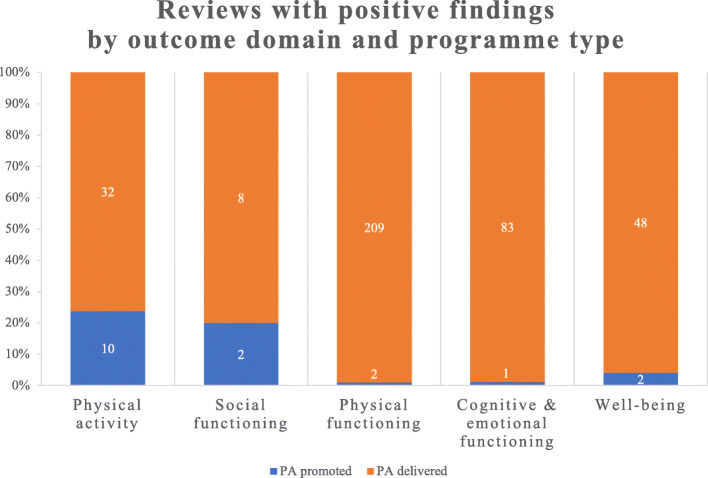
Systematic reviews of physical activity programmes for older adults with positive findings by outcome domain and programme type. Note: ‘PA promoted’ refers to interventions such as health education, coaching and physical activity referral designed to increase total levels of physical activity. These interventions are often measured by physical activity outcomes such as minutes/week of moderate-vigorous physical activity, total metabolic output and/or total steps. ‘PA delivered’ refers to interventions, such as structured exercise like strength, balance, functional and/or resistance training, where the activity is provided directly to the participant. Therefore, this figure summarises the reviews reporting a positive finding in this outcome domain. Positive findings in more than one outcome domain are possible. The data labels indicate the number of reviews reporting a positive finding in that outcome category

The majority of reviews (290/342, 85%) reported at least one positive finding, in favour of intervention(s), in at least one of the five outcome domains of interest. Although, positive effects were most commonly reported for physical and cognitive/emotional functioning outcomes. Most reviews were of programmes that were delivered physical activity directly to participants, some with education/promotion/coaching components, and measuring physical functioning outcomes (Table 3 and Fig. 3). Fewer reviews evaluated the effect of physical activity promotion alone with overall physical activity as an outcome. Some reviews evaluated intervention(s) effects on quality of life/wellbeing but very few assessed outcomes of social participation or functioning.

The majority of physical activity programmes were structured exercise such as strength, balance, functional and resistance training, or physical recreation such as yoga, tai chi and walking, or mixed activities. Very few reviews considered sporting interventions for older adults and none used sport as a primary eligibility criterion. Most interventions were delivered by health and/or exercise professionals. Almost no reviews reported on programmes delivered by volunteers or carers, although a few assessed programmes ***for*** carers with outcomes of emotional functioning. The majority of reviews (259/342, 76%) included studies delivered in multiple locations (mixed) with most of these carried out in community facilities or health services, and many incorporating a home-based component.

Relevant to research question three, there were clear gaps in the evidence regarding reviews of intervention(s) with large-scale population-based study designs, particularly in mass media, social media, and environmental intervention(s). While there was some coverage of participants in residential care, included reviews overwhelmingly targeted community-dwelling older adults living in their own home. Similarly, there were very few reviews of physical activity interventions for older people living in rural or regional settings or with cultural or financial disadvantage, such as migrant or indigenous populations. Very few reviews considered health economic or large-scale implementation criteria.

While gender breakdown was not calculated at the level of individual studies in this scoping review, there was only one review explicitly targeting one gender, females. Samples were still predominantly female but most reviews did not exclude based on gender. There was a mix of individual and group-based interventions but only a few reviews that considered the benefits in terms of social participation of group-based interventions. This was also reflected in the lack of reviews on physical activity interventions in faith-based settings, sporting interventions and explicitly outdoor or park-based settings. There was also no clear focus on the tailoring/adaptation of existing community-based physical activity to include older adults with other age groups.

Devices were used, although mostly by professionals in the measurement of outcomes in the main intervention(s) rather than intended for long-term, mass engagement by participants. The only exception to this was the use of exergames which featured in the eligibility criteria of several reviews with positive effects on physical functioning outcomes. There was a gap in the clear definition of comparators, many were passive such as education, and most were mixed active/passive at the review level making comparisons of format (individual/group) and efficacy difficult.

## Discussion

This scoping review found modest evidence over a 10-year period for research question one with 39 reviews of intervention(s) designed to enhance total physical activity in older adults. The majority (30/39, 77%) of these reported positive overall findings, others were inconclusive. All reviews included for research question one were categorized as meeting *GAPPA Objective 3. Active People* and most assessed physical activity programmes [32, 38, 44, 55] for older adults with some attention to health promotion/coaching [37, 52] and health professional education/referral [30, 56]. This demonstrates a lack of large-scale, population health-based study designs with long term follow-up and physical activity outcomes for older adults. It also reflects a broad cultural bias when considering physical activity and older people. At the time of writing, even WHO’s own Global Health Observatory (GHO) data repository does not report the prevalence indicator of insufficient physical activity separately for older adults compared to all adults aged 18 + [61]. This reflects a much-needed paradigm shift for public attitudes on health, ageing and physical activity.

We found an important gap in the evidence regarding research question one, reviews aimed at increasing total physical activity in older adults, for *GAPPA 1. Active Societies* (mass media, social media and other campaigns) and *2. Active Environments* (interventions changing the environment) objectives. While it is possible that our search terms and scoping review methodology did not address these objectives as exhaustively as they did for *GAPPA 3. Active People,* it is more likely that the reviews are lacking because there are insufficient individual intervention studies. This is consistent with other research concluding that physical activity is ‘good for older adults’ but that programme implementation is being overlooked [62]. It suggests a bias towards short-term interventional studies in controlled settings with selected samples, and away from population-based and large-scale physical activity research. Similarly, for *GAPPA 4. Active Systems*, there were many eHealth, digital and activity trackers reviews [29, 31, 34, 45, 47, 50, 51, 57, 59, 60, 63] but none exploring monitoring at population or systemic levels. Further investigation is now required to identify individual studies, including unpublished data, in these areas.

In research question two, most (290/342, 85%) reviews of physical activity programmes and services reported at least one positive finding in an outcome domain of interest, most commonly physical activity [23, 26, 42], physical [64–66] and cognitive/emotional functioning [67–70], supporting the effectiveness of these interventions for older adults. However, there is far less research targeting outcomes of social functioning [71, 72] and well-being/quality of life [73–75]. This will require a necessary shift to a population-based approach in physical activity research for older adults.

Consistent with recent literature [12], most of the reviews we identified relate to interventions of structured exercise [76, 77], including strength, balance, and resistance training, and physical recreation [78–80] including yoga and tai chi. A number of structured exercise interventions included video or exergames [81–83] but no reviews targeted sport-based programmes for older adults and this should be investigated further. Many reviews also failed to be selective for intervention type, mixing structured exercise and physical recreation, making recommendations about efficacy/acceptability difficult.

In line with our inpatient exclusion criterion, most eligible reviews targeted community-dwelling older adults [9, 84], although there was some coverage of residential care settings [41, 85], particularly in the falls prevention literature. Despite many older people maintaining employment, there were no reviews exclusively targeting the workplace and only two including a workplace setting [25, 86] and one of physical activity in ‘retirement transition’ [26].

Importantly, few of the reviews in either research question appeared to consider the diversity of function and intrinsic capacity in older adults. There were very few reviews focused on the ‘oldest old’ [87], and samples from geographically [49] or socioeconomically [43, 48, 88] disadvantaged populations and none targeted low-income countries. This is likely due to the lack of underlying longitudinal studies of these samples but also reveals inherent difficulties in the recruitment and retention of older adults [89, 90]. Moreover, many older adults are living with chronic/comorbid conditions and preconditions that confound studies of ‘healthy’ samples. Although we excluded reviews of specific medical conditions, review selection criteria of ‘healthy older adults’ was common [67, 74, 80], despite the high prevalence of chronic comorbidity.

### Strengths and limitations

The comprehensive search strategy, the GAPPA, and two-tier coding framework based on TIDieR, PROFANE, and the ICF were major strengths of this review. This review analysed a large and encouraging volume of research of physical activity interventions for older people and identified important gaps in the literature.

A scoping review methodology, such as that adopted in this study, is limited in its capacity to make recommendations about the efficacy of individual interventions. Reviews investigating the effects of physical activity interventions combined with other interventions were excluded. This may have resulted in the omission of important/relevant evidence regarding multicomponent or ‘real-world’ interventions. Commensurate with a scoping methodology, the scale of this review did not permit quality assessment at the level of individual studies. As this analysis remained at the review level, not the level of individual studies, it is also possible that studies were counted more than once across multiple reviews. These should be addressed in a subsequent review of individual studies.

## Conclusion

This scoping review identified a modest volume of evidence regarding interventions designed to increase total physical activity in older people (research question one), although more population-based and long-term follow-up intervention studies are needed, particularly in health promotion, coaching and health professional education/referral between healthcare and community settings. Research gaps were identified in social (mass-media), environmental, and systemic areas for the GAPPA objectives other than 3. Active People.

This review also found a large amount of evidence regarding specific physical activity programmes and services (research question two). In particular, there was a substantial volume of evidence demonstrating benefits of structured exercise and to a lesser extent physical recreation, particularly on outcomes of mobility, strength and balance. However, we found gaps in studies of sporting and workplace physical activity interventions targeting older people. There was also a notable lack of research in diverse/disadvantaged samples and social participation outcomes. The next steps are to assess each of the individual studies covered in these reviews and initiate research to address the gaps in evidence. These represent opportunities for an evidence base that more closely reflects the diversity of ageing with widespread acceptance of the right to the health and social benefits of physical activity at every age.

## Data Availability

The datasets used and analysed during this review are available from the authors on reasonable request.

## Abbreviations

CINAHL: Cumulative Index to Nursing and Allied Health Literature
CONSORT: Consolidated Standard of Reporting Trials
GAPPA: Global Action Plan on Physical Activity (WHO) [7]
GSAP: Global Strategy and Action Plan on Ageing and Health (WHO) [2]
ICF: International Classification of Functioning, Disability and Health [15]
NCD: Noncommunicable diseases
PA: Physical activity
PEDro: Physiotherapy Evidence Database (www.pedro.org.au)
PICO: Population, Intervention, Comparator, Outcome
PRISMA-ScR: PRISMA Extension for Scoping Reviews [20]
PROFANE: Prevention of Falls Network Europe
TIDieR: Template for Intervention Description and Replication (CONSORT)
WHO: World Health Organization
